# Hofmeister Effect in RT-QuIC Seeding Activity of Chronic Wasting Disease Prions

**DOI:** 10.3389/fbioe.2021.709965

**Published:** 2021-09-30

**Authors:** Soyoun Hwang, Danielle Beckley, Konstantin P. Alekseev, Eric M. Nicholson

**Affiliations:** ^1^ United States Department of Agriculture, Agricultural Research Service, National Animal Disease Center, Virus and Prion Research Unit, Ames, IA, United States; ^2^ U.S. Department of Energy, Oak Ridge Institute for Science and Education, Oak Ridge, TN, United States; ^3^ N. F. Gamaleya National Research Center for Epidemiology and Microbiology, Moscow, Russia

**Keywords:** Hofmeister series, salt ions, prion diseases, chronic wasting disease (CWD), RT-QuIC, diagnostics, seeding activity, transmissible spongiform encephalopathy

## Abstract

Chronic wasting disease (CWD) is a transmissible spongiform encephalopathy (TSE) that causes a fatal neurodegenerative disease in cervids. Cases of CWD are rapidly increasing in North America among wild and farmed cervid populations, and potential for zoonotic transmission is not yet determined. Therefore, in order to manage the disease, it is imperative to devise a system that can detect CWD during its early phases to prevent spread to new captive herds through introduction of CWD-affected animals into otherwise CWD-free herds. Real-time quaking–induced conversion (RT-QuIC) assays have been applied to detect the presence of disease-associated prions from various samples in both animals and humans. In this study, we have tested the use of five Hofmeister anions that range from weakly hydrating to strongly hydrating: Na_3_citrate, Na_2_SO_4_, NaCl, NaI, and NaClO_4_ in RT-QuIC reactions for CWD seeding activity using different recombinant prion proteins as substrates. This work shows how the ionic environment of the RT-QuIC reaction can enhance or diminish the seeding activity. The use of Na_2_SO_4_ or NaI as the sodium salt for RT-QuIC using bank vole recombinant prion substrate for the detection of CWD using brain samples reduces the lag time to detect with reasonable specificity. For detection of the CWD in fecal samples, only NaI showed comparable reduction in lag time relative to NaCl but required reduced temperature to alleviate spontaneous fibril formation in negative control samples. Selection of the proper ion environment and recombinant prion protein substrate will make RT-QuIC a powerful diagnostic tool for early detection of CWD prions, further supporting CWD surveillance in wild and captive cervids.

## Introduction

Prion diseases, also called transmissible spongiform encephalopathies (TSEs), are a class of fatal neurodegenerative diseases affecting humans and animals, some of which are zoonotic. They include Creutzfeldt–Jakob disease (CJD) in humans, bovine spongiform encephalopathy (BSE) in cattle, scrapie in sheep and goats, and chronic wasting disease (CWD) in cervids (Prusiner, 1982; Prusiner, 1998; Collinge, 2001; Cheng et al., 2016). Different TSEs have different effects on their hosts, but universally, they cause neuronal death in the central nervous system that leads to serious neurological clinical presentation, such as changes in behavior, loss of motor control, ataxia, and eventually death (Imran and Mahmood, 2011). In most TSEs, misfolded proteins predominantly accumulate in the central nervous system with more limited lymphoid accumulation. In CWD, the lymphoid accumulation can be extensive with reports indicating detectable accumulation in muscle and blood (Prusiner, 1982; Collinge, 2001; Cheng et al., 2016). It has also been shown that CWD-infected animals shed prions via saliva, urine, and feces. Due to this shedding, CWD easily spreads in both captive and free-ranging cervids. However, there is a lack of effective CWD management strategies to stop the spread, which indicates that the management will rely on early detection of prions in CWD-infected animals.

A highly sensitive and specific method that tests for the presence of infectious prions (PrP^Sc^) in various samples from humans and animals is real-time quaking–induced conversion (RT-QuIC), which assesses pathogenic prion seeding activity using a recombinant prion protein substrate (rPrP) (Wilham et al., 2010; Atarashi et al., 2011; Orru et al., 2012; Cheng et al., 2014; Orrú et al., 2014; Orrú et al., 2015a; Orrú et al., 2015b). The success of an RT-QuIC assay depends on the balance of two competing forces: the test’s sensitivity and the test’s specificity (Cheng et al., 2014). To make the RT-QuIC test more sensitive, experimental conditions can be altered to make the rPrP substrate more likely to form the detectable amyloid fibril during the assay. These alterable conditions include temperature, pH, salt concentration, detergent, genotype of rPrP substrate, and dilution of seed (Cheng et al., 2014; Orrú et al., 2014; Orrú et al., 2016).

Salt is a necessary part of the reaction mixture used in RT-QuIC. Most laboratories use NaCl as their salt of choice, but a recently published article showed that the influence different salts have when combined with different seeds and substrates (Metrick et al., 2019). The study investigated the trial of several different Hofmeister series salts in RT-QuIC assays designed to detect protein-misfolding diseases including Alzheimer’s disease, Parkinson disease, and prion disease. The Hofmeister series, devised by 19th century protein scientist Franz Hofmeister, are lists of salts ions that rank the ion on its relative ability to “salt-in” or “salt-out” proteins from a solution. A protein is salted in when the interaction between the charge of the salt ions and the charge of the different regions of the protein enhances solubility, and the protein is salted out when the charges cause the protein to precipitate (Crevenna et al., 2012; Light et al., 2016). It will be useful to continue the investigation into the effects of the Hofmeister ions on seeding activity, focusing on the seeding activity of CWD samples prepared from deer brain and feces.

In this study, we chose a selection of Hofmeister ions that were previously used by Metrick et al. (Na_3_citrate, Na_2_SO_4_, NaCl, NaI, and NaClO_4_), and we optimized the concentration of these salts by titration for CWD prion detection using RT-QuIC. Since the previous study showed significantly different seeding activity of human samples depending on the seeds used and the substrate choice, we also tested two different substrates in the presence of the different salt ions and CWD samples. We aim to find the optimal conditions to use when testing CWD-infected white-tailed deer samples with RT-QuIC assays.

## Materials and Methods

### Ethics Statement

Archived tissue samples used as RT-QuIC seed in this study were obtained from animal experiments reviewed and approved by the National Animal Disease Center’s Institutional Animal Care and Use Committee (protocol numbers: 3,451 and 3,669 (Hwang et al., 2020), ARS-2017-629 (Hwang et al., 2018a), ARS-2018-748 (Hwang et al., 2021)). The animal experiments were carried out in accordance with the Guide for the Care and Use of Laboratory Animals (Institute of Laboratory Animal Resources, National Academy of Sciences, Washington, DC). The details of this study are described in citation numbers 17, 18, and 19.

### Sources of RT-QuIC Seed

Archived brain samples and fecal samples from CWD-infected white-tailed deer and sheep were obtained from studies (Hwang et al., 2018a; Hwang et al., 2020; Hwang et al., 2021) previously conducted at the National Animal Disease Center. For this work, from the aforementioned studies, we used 1 CWD-positive white-tailed deer brain, 1 scrapie positive sheep brain, 1 negative control white-tailed deer brain, 2 CWD-positive fecal samples from white-tailed deer, and 1 negative control fecal sample from white-tailed deer. All samples were frozen at −80 °C upon collection and stored until thawed for analysis.

### Recombinant Prion Protein Production and Purification

*E. coli* (BL21 (λDE3)) was transformed with the pET28a vector containing the BV PrP gene (amino acids 23–231; GenBank accession number AF367624) and bovine PrP (amino acids 25–241; GenBank accession number: DQ875147.1). The recombinant prion proteins were expressed and purified, as described by Vrentas *et al* (Vrentas et al., 2012). The concentration of pooled protein eluent was measured by UV and calculated from the absorbance at 280 nm using an extinction coefficient of 62,005 or 63,495 M^−1^cm^−1^ as calculated for BV (23-231) and bovine (25-241) rPrP (Hwang et al., 2017; Hwang et al., 2018b).

### RT-QuIC Protocol

RT-QuIC reactions were performed, as previously described (Atarashi et al., 2011; Cheng et al., 2014; Orrú et al., 2015a; Orrú et al., 2015b; Dassanayake et al., 2016; Masujin et al., 2016; Orrú et al., 2016; Hwang et al., 2017). The reaction mix was composed of 10 mM phosphate buffer (pH 7.4), 100–500 mM Na_3_citrate, Na_2_SO_4_, NaCl, NaI, or NaClO_4_, 0.1 mg/ml recombinant BV or bovine prion proteins, 10 µM thioflavin T (ThT), and 1 mM ethylenediaminetetraacetic acid tetrasodium salt (EDTA). For Hofmeister ion comparisons, 400 mM of each was used. Aliquots of the reaction mix (98 µL) were loaded into each well of a black 96-well plate with a clear bottom (Nunc, Thermo Fisher Scientific) and seeded with 2 µL of brain homogenate or feces dilution from 10^−1^ to 10^−10^ of a 10% (w/v) stock. The plate was then sealed with a plate sealer film and incubated at 42°C (brain) or 37°C (feces) in a BMG FLUOstar Omega plate reader with cycles of 1 min shaking (700 rpm double orbital) and 1 min rest for 100 h. ThT fluorescence measurements (excitation, 460 nm; emission 480 nm, bottom read, 20 flashes per well, manual gain 1,400) were taken every 45 min.

All reactions for each dilution and each sample were performed in four replicates of RT-QuIC assays. ThT fluorescence data are displayed as the average ThT fluorescence of four technical replicates for each time point and, to be considered positive, the ThT fluorescence of at least two replicate reactions must be positive. As previously described for classification of positive samples by RT-QuIC, the positive threshold was calculated as the mean value of fluorescence from wells containing non-inoculated control white-tailed deer brain or feces homogenates plus 10 standard deviations (Orrú et al., 2014; Orrú et al., 2015c; Dassanayake et al., 2016). Data are reported as time to threshold, also referred to as lag time, of each set of four replicates.

## Results

### Hofmeister Ion Effects on RT-QuIC Reactions Seeded With Brain Material From CWD-Infected White-Tailed Deer Using Recombinant Bank Vole (BV) PrP Substrate

**FIGURE 1:**
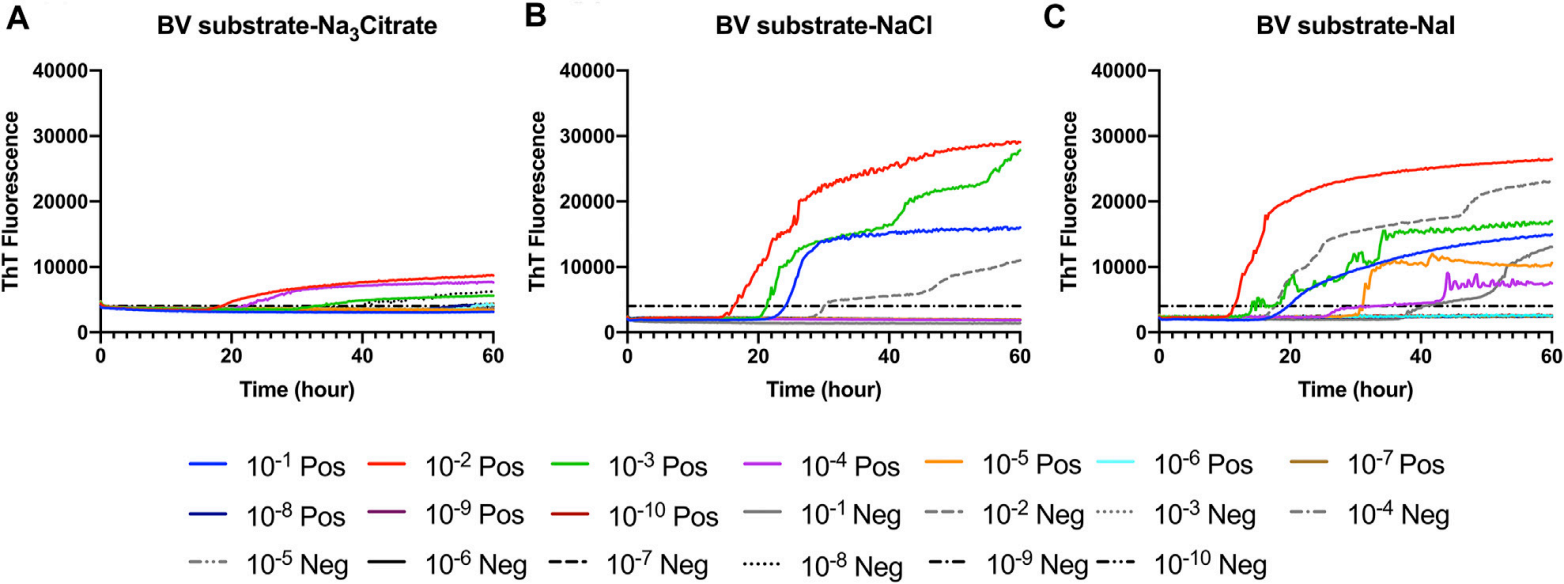
Representative Hofmeister ion effects on RT-QuIC reactions seeded with CWD-positive white-tailed deer brains using the BV rPrP substrate. RT-QuIC reactions were seeded with 10^−1^, 10^−2,^ 10^−3^, 10^−4,^ 10^−5^, 10^−6^, 10^−7^, 10^−8^, 10^−9^, and 10^−10^ dilutions of brain homogenate from one CWD-positive and one CWD-negative white-tailed deer with the addition of 0.001% of SDS. Substrate contained bank vole rPrP and 400 mM Na_3_citrate **(A)**, NaCl **(B)**, or NaI **(C)**. Reactions were run at 42°C. Data are presented as a mean ThT fluorescence of four repeated reactions.

**FIGURE 2:**
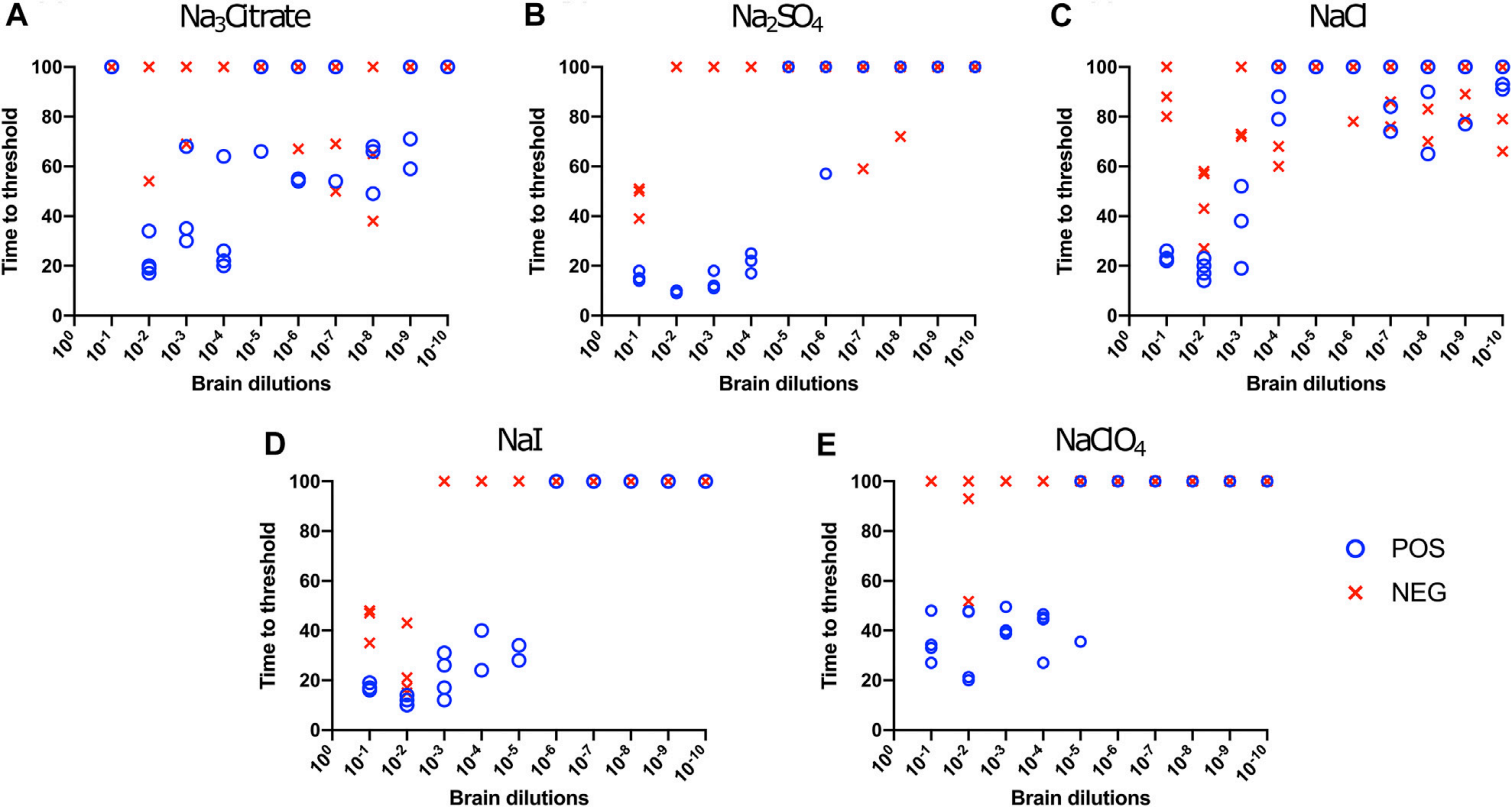
Hofmeister ion effects on RT-QuIC reactions seeded with CWD-positive white-tailed deer brains using BV rPrP substrate. RT-QuIC reactions were seeded with 10^−1^, 10^−2,^ 10^−3^, 10^−4,^ 10^−5^, 10^−6^, 10^−7^, 10^−8^, 10^−9^, and 10^−10^ dilutions of brain homogenate from one CWD-positive and one CWD-negative white-tailed deer with the addition of 0.001% of SDS. Substrate contained bank vole rPrP and 400 mM Na_3_citrate **(A)**, Na_2_SO_4_ **(B)**, NaCl **(C)**, NaI **(D)**, or NaClO_4_ **(E)**. Reactions were run at 42°C.

First, we investigated Hofmeister ion effects on RT-QuIC reactions prepared with recombinant BV prion protein substrate and seeded with the brain material from CWD-infected white-tailed deer (WTD). To determine the effect of an ion’s rank in the Hofmeister series has on RT-QuIC seeding activity, each reaction containing one of three representative Hofmeister salts—NaI (weakly hydrating), NaCl, (moderately hydrating), or Na_3_citrate (strongly hydrating)—was seeded with brain dilutions (10^−1^ to 10^−10^) from CWD-infected white-tailed deer. As illustrated in Figure 1, the overall RT-QuIC reactions showed seeding activity for the less diluted brain dilutions in the presence of these salts, while still allowing for discrimination of those assays seeded with negative brain. However, assays containing Na_3_citrate exhibited relatively low ThT fluorescence compared to assays containing the other two salts. RT-QuIC assays containing NaI shortened the time to threshold and also enhanced the detection sensitivity while still allowing for discrimination between assays seeded with positive and negative samples. In addition to the three representative ions, we also tested two more salts, Na_2_SO_4_ (weakly hydrating) and NaClO_4_ (strongly hydrating), to further evaluate the Hofmeister ion effect. As shown in Figures 2B,D, assays containing Na_2_SO_4_ appear very similar to assays containing NaI. Both salts create shorter time to threshold (within 20 h), specifically for reactions seeded with brain dilutions of 10^−1^, 10^−2^, 10^−3^, and 10^−4^. Also, all the assays seeded with positive samples showed shorter time to threshold than assays seeded with negative samples maintaining the ability to discriminate positive from negative samples. Assays containing NaClO_4_ also showed good seeding activity, but the time to threshold is relatively longer than reactions containing Na_2_SO_4_ or NaI. Based on these results, RT-QuIC reactions that combined Na_2_SO_4_ or NaI with BV substrate are most likely to allow for sensitive and specific detection of CWD prions.

### Hofmeister Ion Effects on RT-QuIC Reactions Seeded With Brain Material From CWD Infected White-Tailed Deer Using Recombinant Bovine PrP Substrate

**FIGURE 3:**
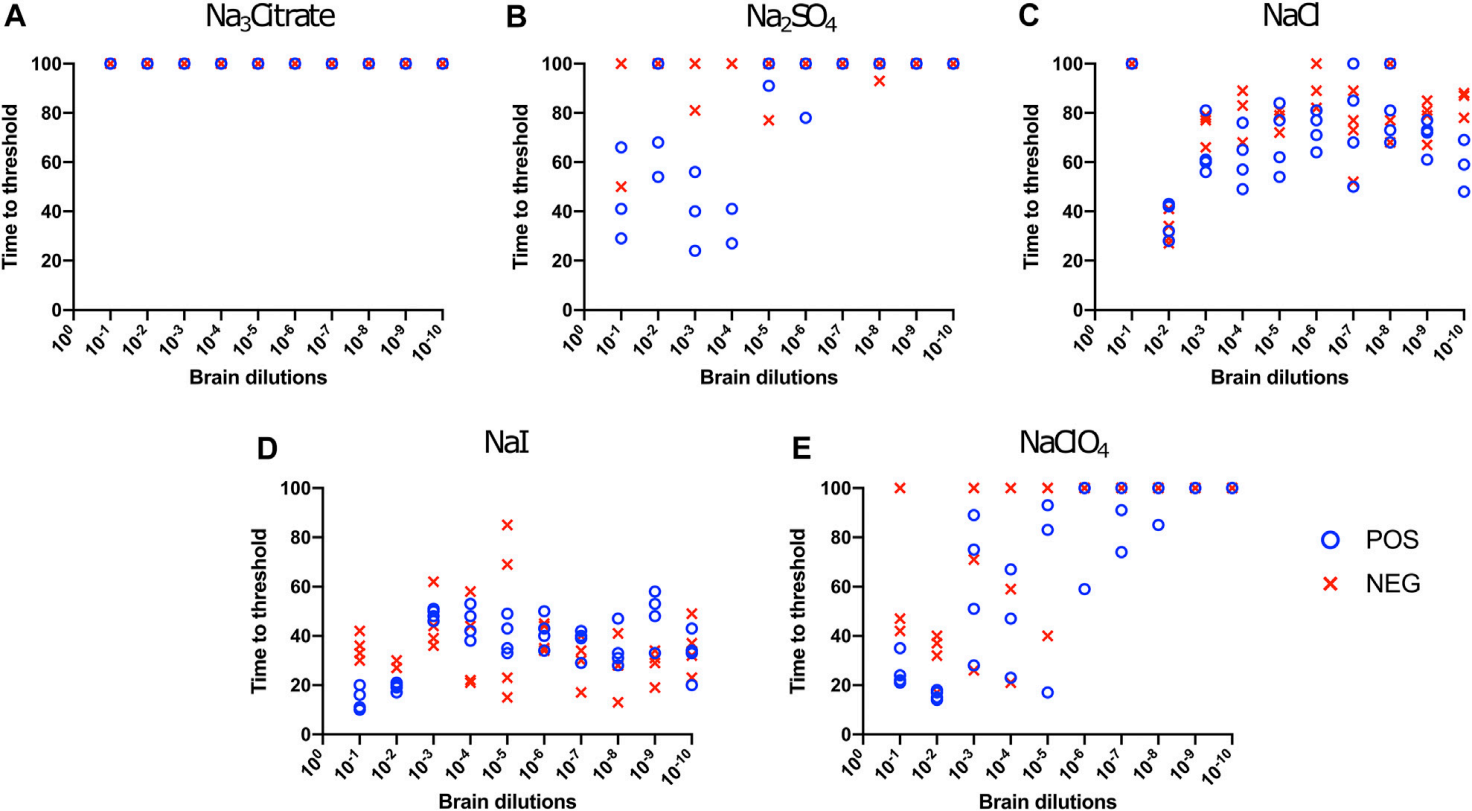
Hofmeister ion effects on RT-QuIC reactions seeded with CWD-positive white-tailed deer brains using bovine rPrP substrate. RT-QuIC reactions were seeded with 10^−1^, 10^−2,^ 10^−3^, 10^−4,^ 10^−5^, 10^−6^, 10^−7^, 10^−8^, 10^−9^, and 10^−10^ dilutions of the brain homogenate from one CWD-positive and one CWD-negative white-tailed deer with the addition of 0.001% of SDS. Substrate contained bovine rPrP and 400 mM Na_3_citrate **(A)**, Na_2_SO_4_ **(B)**, NaCl **(C)**, NaI **(D)**, or NaClO_4_ **(E)**. Reactions were run at 42°C.

We also evaluated the influence of Hofmeister anions on RT-QuIC reactions when using recombinant bovine prion protein. Each reaction containing with the salts Na_3_citrate, Na_2_SO_4_, NaCl, NaI, and NaClO_4_ was seeded with brain dilutions (10^−1^ to 10^−10^) from CWD-infected white-tailed deer and negative control animals. In general, compared to the BV substrate, assays with bovine substrate showed lower seeding activity based on an increase in time to threshold for positive samples and a reduced ability to discriminate negative and positive samples due to a decrease in time to threshold for negative samples. For example, in the assays containing Na_3_citrate, no samples exhibited increased ThT fluorescence indicative of fibril formation (Figure 3A). The time to threshold for all four positive wells was shorter than that for all four negative wells only for the reaction mixture containing Na_2_SO_4_ seeded with 10^−3^ and 10^−4^ brain dilutions (Figure 3B), and just barely so for the NaI substrate seeded with 10^−1^ and 10^−2^ brain dilutions (Figure 3D) and the NaClO_4_ substrate seeded with 10^−1^ brain seed (Figure 3E). Therefore, RT-QuIC assays containing NaI and seeded with low brain dilutions are most likely to allow for a successful detection and discrimination between positive and negative seeds when tested with bovine substrate. Based on our results, though, the use of the bovine substrate at all is not preferable for CWD prion detection.

### Effect of Titration of Sodium Salts on RT-QuIC Reactions With Recombinant BV Substrate

**FIGURE 4:**
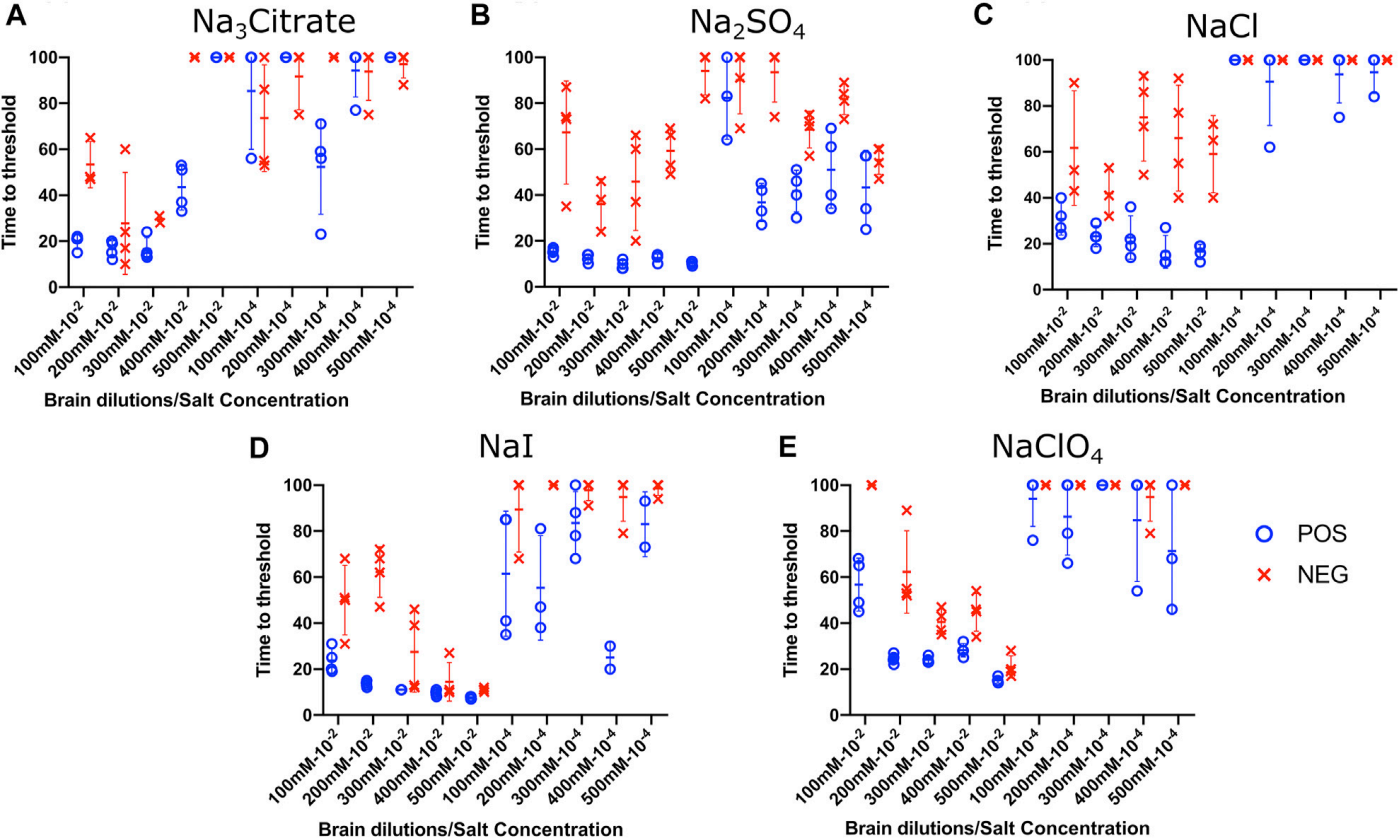
Effect of titration of Hofmeister ions on RT-QuIC reactions seeded with CWD-positive white-tailed deer brains using BV rPrP substrate. RT-QuIC reactions were seeded with 10^−2^ (left side of each graph) and 10^−4^ (right side of each graph) dilutions of the brain homogenate from one CWD-positive and one CWD-negative white-tailed deer with the addition of 0.001% of SDS. Substrate contained BV rPrP and Na_3_citrate **(A)**, Na_2_SO_4_ **(B)**, NaCl **(C)**, NaI **(D)**, or NaClO_4_ **(E)**. The concentration of each salt was tested at 100, 200, 300, 400, and 500 mM. Reactions were run at 42°C.

To evaluate the influence of salt concentration on the seeded conversion of the BV rPrP substrate with CWD agent, two different dilutions (10^−2^ and 10^−4^) of brain tissues were used to seed RT-QuIC reactions prepared with the same five salts (Na_3_citrate, Na_2_SO_4_, NaCl, NaI, and NaClO_4_), this time at increasing concentrations: 100, 200, 300, 400, and 500 mM. Overall, the less diluted 10^−2^ brain seeds performed better at shortening time to threshold than the 10^−4^ brain seeds did (Figure 4) presumably due to the presence of more CWD prions to function as seed in the reaction. Reactions with different salts reacted differently to changes in concentration. For example, RT-QuIC assays containing Na_3_citrate yielded better seeding activity with lower concentrations of salt, 100–300 mM, than reactions containing higher concentrations, 400 or 500 mM. Other than these reactions with Na_3_citrate, all other reactions showed shorter time to threshold with high salt concentrations. When any concentration of Na_2_SO_4_, especially the highest 500 mM concentration, was used in the BV substrate, it allowed all the positive wells to have a short time to threshold, mostly within 10 h. These reactions also exhibited good discrimination (Figure 4B). It is also important to note that RT-QuIC reactions seeded with 10^−4^ brain dilution containing higher concentrations of NaI and Na_2_SO_4_ still show seeding activity and clear discrimination between positive and negative samples.

### Effect of Titration of Sodium Salts on RT-QuIC Reactions With Recombinant Bovine Substrate

**FIGURE 5:**
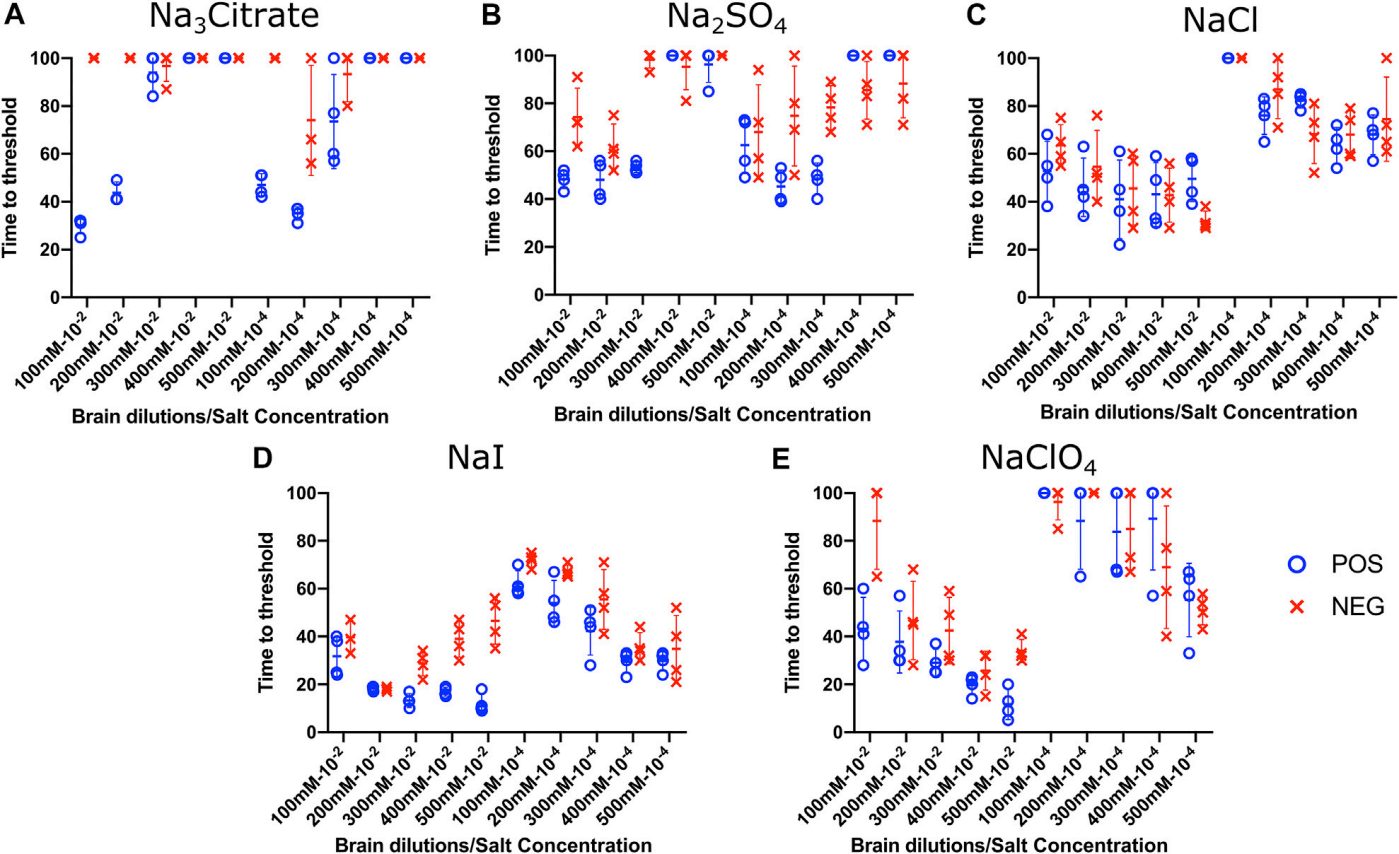
Effect of titration of Hofmeister ions on RT-QuIC reactions seeded with CWD-positive white-tailed deer brains using bovine rPrP substrate. RT-QuIC reactions were seeded with 10^−2^ (left side of each graph) and 10^−4^ (right side of each graph) dilutions of brain homogenate from one CWD-positive and one CWD-negative white-tailed deer with the addition of 0.001% of SDS. Substrate contained bovine rPrP and Na_3_citrate **(A)**, Na_2_SO_4_ **(B)**, NaCl **(C)**, NaI **(D)**, or NaClO_4_ **(E)**. The concentration of each salt was tested at 100, 200, 300, 400, and 500 mM. Reactions were run at 42°C.

To evaluate the influence of salt concentration on the seeded conversion of the bovine rPrP substrate with the CWD agent, two different dilutions (10^−2^ and 10^−4^) of brain tissues were used to seed RT-QuIC reactions prepared with the same five salts (Na_3_citrate, Na_2_SO_4_, NaCl, NaI, and NaClO_4_) at increasing concentrations: 100, 200, 300, 400, and 500 mM. As seen in the BV RT-QuIC results, a 10^−4^ dilution of brain seed generally caused longer time to threshold for positive and negative wells than a 10^−2^ dilution did. Overall, RT-QuIC reactions with bovine substrate showed longer time to threshold than reactions with BV substrate. As shown in Figure 5, RT-QuIC assays with recombinant bovine substrate reacted differently depending on salt concentrations and salt type. For example, reactions in the presence of Na_3_citrate or Na_2_SO_4_ yielded shorter time to threshold for low salt concentrations, 100 and 200 mM, while reactions in the presence of NaI or NaClO_4_ yielded shorter time to threshold for high salt concentrations, 300, 400 and 500 mM. Assays with NaCl, the default RT-QuIC salt, did not allow for discrimination between CWD-positive and -negative samples using the recombinant bovine substrate. For the purpose of discrimination using the bovine substrate, Na_3_citrate is the best choice because this is the only salt that gives very clear discrimination for both 10^−2^ and 10^−4^ dilutions of brain samples, although the assays exhibit an extended time to threshold. For the purpose of fast detection of CWD prion with recombinant bovine protein, 400–500 mM of NaI is the best salt to use, but again, the bovine protein is poor choice of substrate for CWD detection.

### Hofmeister Ion Effects on RT-QuIC Reactions Seeded With Brain Dilutions From Scrapie-Infected Sheep Using Recombinant BV Substrate

**FIGURE 6:**
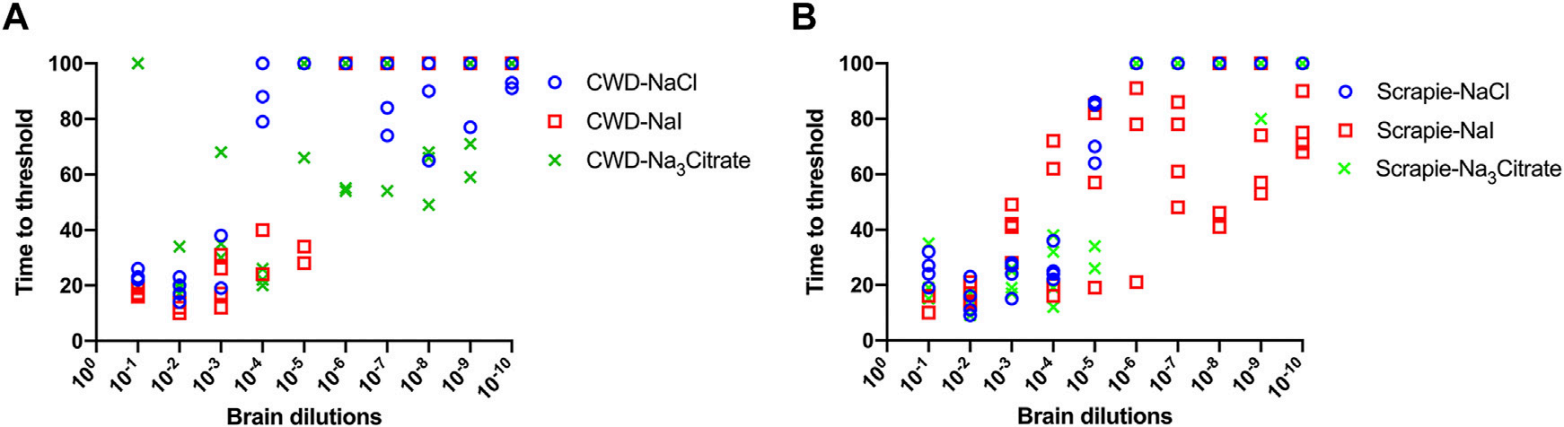
Representative Hofmeister ion effects on RT-QuIC reactions seeded with scrapie-positive sheep brains using BV rPrP substrate. RT-QuIC reactions were seeded with 10^−1^, 10^−2^, 10^−3^, 10^−4,^ 10^−5^, 10^−6^, 10^−7^, 10^−8^, 10^−9^, and 10^−10^ dilutions of the brain homogenate from one CWD-positive deer **(A)** and one scrapie-positive sheep **(B)** with the addition of 0.001% of SDS. Substrate contained BV rPrP and 400 mM NaCl, NaI, or Na_3_citrate. Reactions were run at 42°C.

We also evaluated the influence of Hofmeister anions on RT-QuIC reactions prepared with recombinant BV protein and seeded with prion seeds other than CWD. To accomplish this, we used sheep brainstem homogenate from scrapie-infected sheep to seed reactions, as shown in Figure 6. Ten different dilutions (10^−1^ to 10^−10^) of sheep brain tissues were used to seed the RT-QuIC reactions prepared with three representative ions: Na_3_citrate, NaCl and NaI. When the RT-QuIC reactions were seeded with scrapie, NaI improved the sensitivity of the reactions, while it did not have this effect on seeding activity with CWD prions. Based on these results, Hofmeister ions have different effects on RT-QuIC reactions depending on the selected seed.

### Hofmeister Ion Effects on RT-QuIC Reactions Seeded With Fecal Homogenate From CWD-Infected White-Tailed Deer Using Recombinant BV Substrate

**FIGURE 7:**
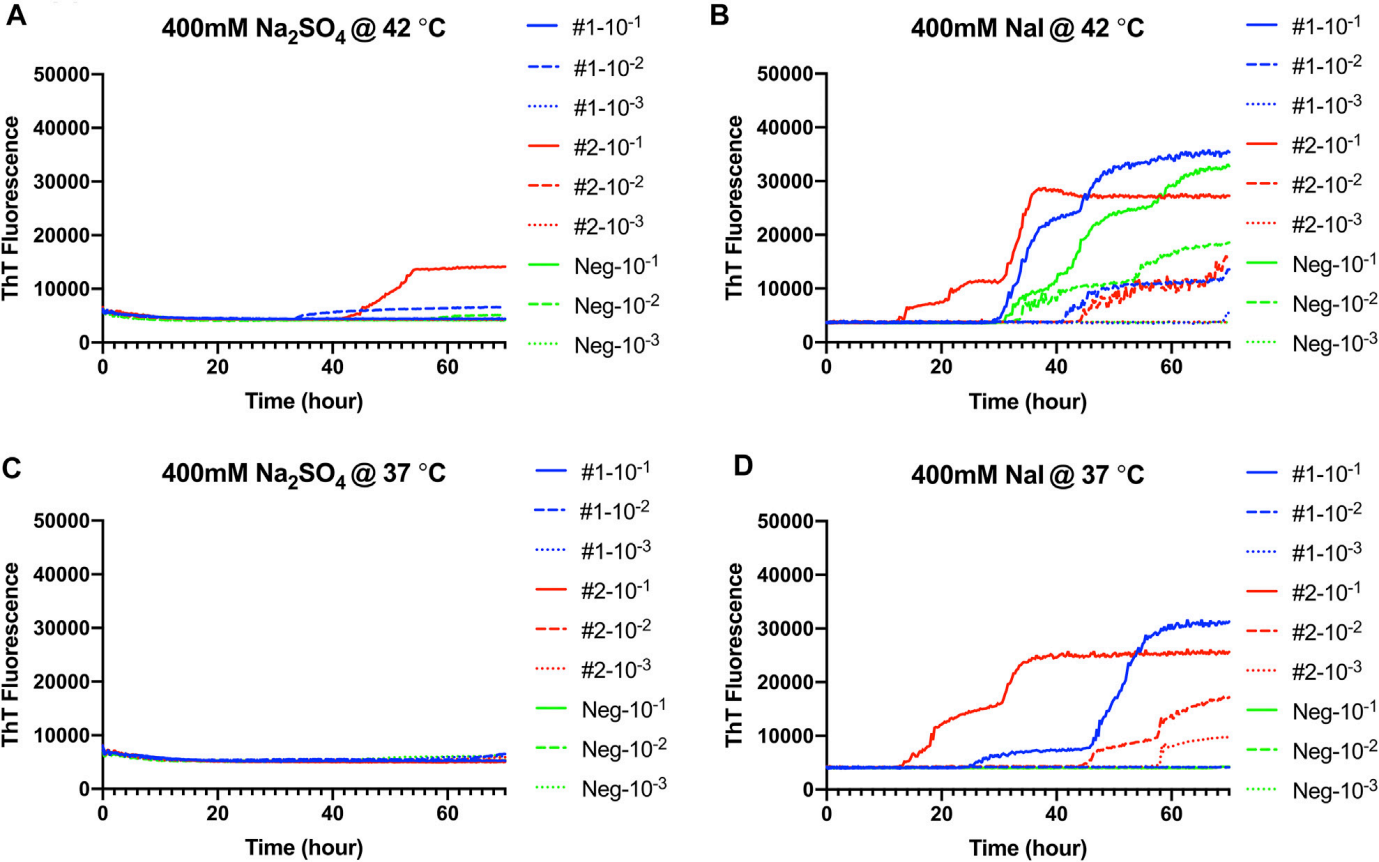
Representative Hofmeister ion effects on RT-QuIC reactions seeded with CWD-positive white-tailed deer feces using the BV rPrP substrate. RT-QuIC reactions were seeded with 10^−1^, 10^−2^, or 10^−3^ dilutions of fecal homogenate from two CWD-positive and one CWD-negative white-tailed deer with the addition of 0.001% of SDS. Substrate contained bank vole rPrP and 400 mM Na_2_SO_4_ **(A,C)** or NaI **(B,D)**. Reactions were run at 42°C **(A,B)** or 37°C **(C,D)**. Data are presented as mean ThT fluorescence of four repeated reactions.

RT-QuIC reaction conditions that had been optimized with recombinant BV prion protein, and CWD-infected brain samples were applied to fecal samples taken from CWD-infected white-tailed deer. Na_2_SO_4_ and NaI were chosen based on assays with brain samples (Figure 7). First, RT-QuIC assays containing either Na_2_SO_4_ (Figures 7A,C) or NaI (Figures 7B,D) were seeded with fecal samples at 42°C. As can be seen in Figure 7, assays containing Na_2_SO_4_ seeded with feces did not result in enhanced seeding activity unlike the assays seeded with the brain homogenate. RT-QuIC assays containing NaI not only exhibit enhanced seeding activity for reactions seeded with positive samples but also exhibit spontaneous conversion when seeded with negative fecal samples and did not allow for discrimination between positive and negative samples. We therefore lowered the assay temperature to 37°C and found that the reduced temperature prevented spontaneous conversion. Na_2_SO_4_ enhanced RT-QuIC reactions seeded with brain samples; however, it did not react the same way with fecal samples even though they are all from CWD-infected white-tailed deer, indicating that the fecal homogenate may need different optimal conditions for better seeding activity in RT-QuIC.

## Discussion

In this study, we analyzed the effects of five Hofmeister anions on the RT-QuIC seeding activity of CWD prions found in the brain material and feces of CWD-infected white-tailed deer. This study shows that those five Hofmeister anions indeed have different effects on seeding activity. While one of these salts, NaCl, has been the default choice for RT-QuIC assays, our study clearly shows that there are more sodium salt candidates that allow higher sensitivity detection of CWD prions by RT-QuIC. Using recombinant BV prion protein, often considered a universal substrate, for RT-QuIC assays, reactions had higher sensitivity and specificity in the presence of NaI or Na_2_SO_4_ than in the presence of NaCl for brain homogenate samples. Our study also found that the effects of the five Hofmeister anions changed when different recombinant prion proteins were used as a substrate. For example, while recombinant bovine prion protein is not an attractive substrate for CWD detection in RT-QuIC reaction mixtures containing NaCl, when a high concentration of NaI is used, the detection of CWD prions is improved. For CWD-affected white-tailed deer fecal samples, regardless of Hofmeister ion used, 37°C remained the optimal temperature as was determined from NaCl. In contrast to the results found for brain homogenates, assays containing Na_2_SO_4_ seeded with feces did not result in enhanced seeding activity. At 42°C, NaI not only exhibited enhanced seeding activity for reactions seeded with positive samples but also exhibited spontaneous conversion when seeded with negative fecal samples and did not allow for discrimination between positive and negative samples; however, lowering the temperature to 37°C recovered the lost specificity. Overall, this study helped our understanding of how salt and substrate selection improves seeding activity of CWD prions found in brains or feces.

In a recently published report, thorough investigation was performed on the effect of Hofmeister ions in the RT-QuIC detection of misfolded proteins related to neurodegenerative diseases in humans including Parkinson’s, Alzheimer’s disease, and prion disease (Metrick et al., 2019). They demonstrated how selection of the proper ionic environment for the assay decreases the difficulty of diagnosing the condition, as even very low amounts of protein aggregates in tissues could be detected as long as the sodium salts and concentrations used in the assays were optimized. They found that different types of misfolded proteins responded differently to ions. For example, tau-based RT-QuIC assays for Alzheimer’s disease and prion-based assays for prion disease worked best in the presence of weakly hydrated anions like Na_3_citrate or Na_2_SO_4_. In our study, we focused on using Hofmeister ions to improve the detection capacity of RT-QuIC when preparing seeds from brain or feces samples from CWD-infected white-tailed deer. While the previous study included assays with CWD prions from ear homogenate seeds, they focused on optimization of tau-based assays for Alzheimer’s seeds and prion-based assays mainly with scrapie. In addition, for the detection CWD, they used recombinant hamster prion protein as a substrate, while recombinant BV prion protein was used in our study. In this study, RT-QuIC assays were run with recombinant BV and bovine prion protein substrate for the detection of CWD prions in brain and fecal homogenates. Both studies suggest that CWD can be detected with better sensitivity in the presence of NaI, a strongly hydrating ion. However, we also found that Na_2_SO_4_, one of the weakly hydrating ions in the Hofmeister series, also improved the CWD detection by shortening detection time. This lack of correlation between the degree of hydration that an ion provides and seeding activity is difficult to interpret with both higher and lower hydrating ions resulting in reduced time to threshold for RT-QuIC–based fibril detection. When the bovine substrate was used, ion hydration did seem to play a role in enhancing CWD detection as our reactions containing strongly hydrating anions like NaI and NaClO_4_ showed shorter time to threshold which allowed for clear discrimination from assays seeded with negative samples. Even though recombinant bovine prion protein is not the best substrate for CWD detection in the presence of NaCl—the typical salt choice, the use of NaI can result in reasonable detection of CWD using bovine recombinant prion protein as a substrate. Thus, depending on the substrate chosen, Hofmeister ions may enhance or diminish an assay’s seeding activity. We also tested the Hofmeister ions with another seed, the homogenized sheep brain from scrapie-infected sheep. Our RT-QuIC assays seeded with scrapie-infected brain homogenate look fairly similar to CWD-seeded samples when made with Na_3_citrate, NaCl, or NaI. Overall, NaI enhanced the RT-QuIC seeding activity with both BV and bovine substrates, reducing the time to threshold to detection while readily allowing discrimination of positive samples from negative samples.

RT-QuIC has been successfully applied for sensitive and specific detection of several protein misfolding diseases, including prion disease. RT-QuIC quickly turned into a powerful tool to assess prion protein distribution in tissues. Even though it is a widely accepted method for research application, it must meet the requirements of reliability and reproducibility to become an approved diagnostic test. Eleven different centers participated in international trials by testing a blind panel of PrP samples using RT-QuIC (McGuire et al., 2016). They used a range of recombinant prion protein substrates and their own, in-laboratory–developed RT-QuIC instrumentations, and the results almost fully coincided among laboratories. Another report showed great agreement for CWD prion detection from rectal biopsies collected from an elk herd using RT-QuIC among all laboratories (Haley et al., 2020). These studies show the potential of RT-QuIC as a powerful diagnostic tool.

In summary, we have tested different Hofmeister anions in RT-QuIC assays to examine their effect on seeding activity of CWD prions. Unlike previous studies on human tau-based assays, we found that CWD was not consistently affected by either strongly or weakly hydrating anions. Instead, when recombinant BV prion protein was used as the substrate, the seeding activity was improved in the reactions containing either NaI (strongly hydrating) or Na_2_SO_4_ (weakly hydrating). This may reflect competing effects on the seed and substrate. However, we did find that when recombinant bovine prion protein was used as a substrate, CWD was detected better in reaction mixtures containing strongly hydrating anions. As the previous study showed, Hofmeister salts have different influences depending on the type of the seed or substrate used, even when the seed features the same prion disease, or the substrates have similar sequences and structures. By focusing on CWD prions, our data aided us in improving RT-QuIC detection of samples taken from CWD-infected white-tailed deer. This approach allows for enhanced detection of CWD in animal tissues or excreta samples that have a low prion concentration. These improvements to disease detection can be applied to future CWD surveillance and control.

## Data Availability

The original contributions presented in the study are included in the article/Supplementary Material; further inquiries can be directed to the corresponding author.
